# Influence of Temperature during Pre-Fermentative Maceration and Alcoholic Fermentation on the Phenolic Composition of ‘Cabernet Sauvignon’ Wines

**DOI:** 10.3390/foods10051053

**Published:** 2021-05-11

**Authors:** Ana Ruiz-Rodríguez, Miguel Palma, Carmelo G. Barroso

**Affiliations:** Center for Agri-Food and Wine Research (IVAGRO), Department of Analytical Chemistry, Faculty of Sciences, University of Cadiz, 11510 Puerto Real, Spain; ana.ruiz@uca.es (A.R.-R.); carmelo.garcia@uca.es (C.G.B.)

**Keywords:** pre-fermentative maceration, alcoholic fermentation temperature, wine making

## Abstract

This study presents the effects of different working temperatures on the transfer of compounds during the pre-fermentative and fermentative stages of the wine making process with ‘Cabernet Sauvignon’ grapes. Two different procedures have been evaluated. Firstly, the pre-fermentative maceration of the crushed grapes at two different temperatures (20 °C and 10 °C). Then, the alcoholic fermentation under two different sets of conditions, the fermentation at a constant temperature of 20 °C and the fermentation under a positive temperature gradient from 10 to 20 °C. According to the experimental results, the phenolic contents (total phenolics, total anthocyanins, and total tannins) were mainly conditioned by the fermentation temperature, however the pre-fermentative conditions also affected the content levels of these compounds. Furthermore, the use of a fermentation temperature gradient improved the organoleptic characteristics of the wines. However, the color was not as stable as that of wines produced through fermentation at a higher constant temperature. Consequently, the implementation of a temperature gradient during the alcoholic fermentation process is recommended and a longer period at high temperature over the last phase of the process would be desirable to obtain aromatic wines with the desirable color stability.

## 1. Introduction

When wine is produced through traditional methods, the grapes’ skin is in contact with the grape juice over the alcoholic fermentation. Winemakers usually keep some control on the extraction conditions that promote the extraction of the components from the solid parts of grapes that are mainly related to wine red color (anthocyanins). Those conditions also affect the extraction of other components (tannins and aroma related compounds). Anthocyanins are easily soluble in water because of their polarity, while tannins are more easily dissolved in must with a certain amount of ethanol content [1,2]. Thus, since wine contains ethanol and has a lower polarity than grape must [3,4], a longer pre-fermentative cold maceration favors the extraction of both anthocyanins and low molecular weight tannins, while a post-fermentation maceration causes mainly the extraction of the tannins that can be found in seeds [5].

With regard to grape aroma related compounds, despite their low polarity they are also extracted from grape skins during the pre-fermentative maceration, especially when low temperature levels are applied in order to prevent the degradation and loss of these compounds [6,7]. However, the effects of pre-fermentative maceration on the recovery of anthocyanins and aroma related compounds depends largely on the grape variety being used [8], its maturity [9], vintage as well as the maceration duration and temperature [10]. Most published papers report a higher extraction of both phenolic and aroma related compounds during cold pre-fermentative maceration; however, some of the published papers inform on some minor effects on the final wines. It has also been reported that this effect may disappear later on over the winemaking processes [11]. It must be noted that the color extraction phenomena related to compounds is also associated to both the degradation of anthocyanins and the condensation of tannins and anthocyanins, which, all in all, make of this a rather complex process [11].

As for the alcoholic fermentation, a continuous solid liquid extraction from the grapes’ skin and seeds takes place. Therefore, the fermentation temperature significantly affects the final composition of the resulting wine [12,13]. Such effect is associated to the compounds extracted from the solid parts of the grapes. In addition, fermentation temperature also has an impact on yeast metabolism, which can be enhanced at high temperatures (above 25 °C). Finally, low temperature fermentation prevents the loss of some of the most volatile compounds [14,15], which are the main ones associated to wine aroma. In this way, low temperature fermentation generally results in more aromatic wines [16], as it increases the concentration of ethyl esters and acetates, and reduces ethanol content [15,17] as long as the temperature is not exceeding Ly low, which may cause deviations from a regular alcoholic fermentation [15,18].

## 2. Materials and Methods

### 2.1. Winemaking

**Figure 1:**
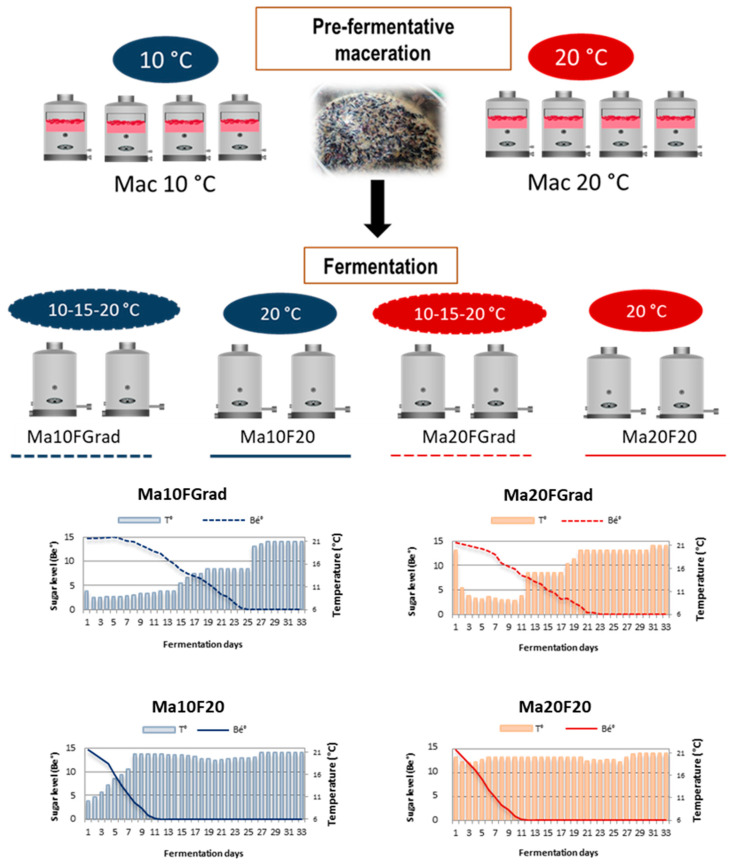
Winemaking conditions: sugar levels and temperature over each step.

Cabernet Sauvignon grapes grown in Jerez (Cadiz, Spain) have been used for this research. The average weight of the berries was 1.20 g (120 g/100 berries). Its sugar levels was 298 g L^−1^ (15 Be) and the value of the total acidity was 4.8 g L^−1^ of tartaric acid equivalents. The grapes were destemmed and crushed. To avoid microbiological activation, sulfur dioxide was added to the pre-treated must up to 30 mg of L^−1^. Tartaric acid (1 g L^−1^) was added to set a pH of 3.6. The pre-fermentation maceration was conducted for 24 h. The four treatments, which have been summarized in Figure 1, were carried out in duplicate. After crushing the grapes, the must and skins were sampled and subjected to different treatments in 50 L tanks (45–48 L per tank): maceration at 10 °C (Ma10FGrad/Ma10F20) and maceration at 20 °C (Ma20FGrad/Ma20F20). The alcoholic fermentation was not started under these conditions, but only after the commercial yeasts were added. After 24 h, the alcoholic fermentation was started under two different conditions: constant 20 °C (Ma10F20/Ma20F20) and under the following temperature gradient: 10 °C until the sugar levels reached 8 °Be, then 15 °C until the sugar level was 3 °Be, and finally 20 °C (Ma10FGrad/Ma20FGrad) (Figure 1).

All the fermentations were carried out using the commercial yeast Viniferm Aura (Agrovin, Alcázar de San Juan, Spain), which contains *Saccharomyces cerevisiae*. It was applied following the recommendations from the supplier. Briefly, 500 g of commercial yeast were added to 5 L of water at 35 °C. After 10 min the mixture was manually shaken for 5 min and then it was let to cool down below 20 °C. Then, 150 mL of this mixture was added to each tank. The supplier recommended the fermentation temperature to be between 12 and 30 °C. This yeast is indicated for young red wines of fresh and floral style. The cap was pierced twice a day throughout the whole process as well as submerged for 10 min before releasing it, no additional aeration was applied. The fermentations were controlled according to the density of the musts.

The wines were then allowed to further settle and cold stabilized at 4 °C for 4 week. Then, 80 mg L^−1^ of sulfur dioxide were added to obtain a free sulfur dioxide concentration between 30 and 40 mg L^−1^ before starting the bottling process. The 8 wine replicas were bottled separately in 750 mL dark amber Bordeaux-style g Lass bottles. All the wines were stored at 20 °C until analysis (2 weeks for the regular parameters and up to one year for the tasting panel).

### 2.2. Analytical Procedures Applied to Musts and Wines

Both the musts and the wines have been characterized by determining their regular parameters (sugar and ethanol contents, pH and total acidity) as well as polyphenols, anthocyanins and total tannins contents. Their density was measured by means of a DMA 4500 M densimeter (Anton Paar, Graz, Austria). The measured density values were used to determine the sugar levels in the musts during their alcoholic fermentation. Their total acidity was determined by acid–base titration using a pHmatic 23 automatic titrator (Crison, Düsseldorf, Germany). Their red color was determined by absorbance measurements at 520 nm by means of a UV/VIS V-530 spectrophotometer (Jasco, Madrid, Spain). Their ethanol content was determined by measuring the density of the distillate by means of the DMA 4500 M densimeter. The distillations were carried out in triplicate.

To determine the anthocyanins and total polyphenols contents in the samples, 1 mL of a 1 M HCl solution was added to 1 mL of each sample, and then the absorbance was measured at 520 and 280 nm respectively by means of a UV/VIS V-530 spectrophotometer (Jasco). The total polyphenols in the samples were expressed as mg gallic acid equivalent to L^−1^. The total anthocyanins were expressed as mg of malvidin-3-g Lucoside equivalent to L^−1^ of the sample. The calibration curves for gallic acid and malvidin-3-g Lucoside were plotted according to seven different levels between 50 and 1000 mg of L^−1^. All the samples were analyzed in duplicate.

In order to determine the total content of tannins, the samples (1 mL) were treated with 0.04% (*w*/*v*) methylcellulose (3 mL) and a saturated solution of ammonium sulfate was added to make up to 5 mL total volume. The absorbance values at 280 nm were compared to those of a water sample treated the same way. The difference between both solutions represented the total concentration of tannins [19]. The total tannins were expressed in mg catechin equivalent to L^−1^. Two catechin calibration curves were generated based on seven different levels from 50 to 1000 mg L^−1^ and from 1000 to 5000 mg L^−1^ respectively.

### 2.3. Descriptive Sensory Analysis

Six months after their bottling, the 8 wines were evaluated by a panel of 12 trained volunteers, formed by six women and six men between 21 and 51 years of age. All of the panel members had some experience in wine tasting and were selected based on their availability and interest. Two training sessions were held in order to set up a series of terminology and reference standards [20]. After the training had been completed, the panelists were requested to evaluate the eight wines in three tasting sessions. In each session, eight wine samples were tasted at random. Six attributes were evaluated in the same order: aromatic intensity, red fruit, floral, pepper, astringency texture and overall rating. The panelists were asked to rate these attributes according to a 0 to 5 scale.

### 2.4. Statistical Analysis

A two-way ANOVA was used to estimate how maceration temperature and the fermentation temperature affected the properties of the musts and wines, i.e., total acidity, pH, ethanol level and absorbance at 520, total phenolics, total tannins, total anthocyanins and color and a X^2^ test was applied to the discrete variable, i.e., the results from the sensory evaluation. The differences were considered as relevant at *p*-value ≤ 0.05. The statistical analyses were carried out by means of SPSS (IBM, Armonk, NY, USA) and Excel (Microsoft, Redmond, WA, USA).

## 3. Results and Discussion

Four wines and their corresponding duplicates were produced under the above described conditions. They were compared based on both the analytical results and the tasting panel’s evaluation. These specific working conditions were selected since many winemakers use 10 °C to lower the temperature of the grapes before starting the alcoholic fermentation and 20 °C is a commonly used temperature for the regular production of red wine.

Before evaluating the effects of the different winemaking techniques on the composition of the wines, it should be noted that, as shown in Figure 1, the duration of the alcoholic fermentation at a constant temperature (Ma10F20/Ma20F20) was only 13 days, while for the fermentation under a temperature gradient (Ma10FGrad/Ma20FGrad), the total process took 27 days. In both cases, the solid material was removed from the liquid after 33 days, therefore the total time the musts/wines were in contact with the cap was 33 days in all the cases. This means that the extraction time was actually the same for all of the winemaking procedures in the experiment. However, the temperature conditions were different for both processes and the liquid medium had different properties, i.e., must vs. wine. Thus, while two of the procedures were run at constant temperature (20 °C) after the alcoholic fermentation had been completed; namely Ma20F20 was kept at 20 °C for 33 days and Ma10F20 was maintained at 20 °C for 26 days (from day 8 to day 33), the samples that were subjected to a temperature gradient reached 20 °C just a few days before the cap was removed as the fermentation process had been completed. This, in the case of Ma20Grad meant from the 20th day to the 33rd day, i.e., 14 days. While in the case of Ma10FGrad the cap was removed from the 27th day until the 33rd day, i.e., 7 days. Since the alcoholic fermentation process of the wines fermented at 20 °C was completed more rapidly, the time that the ethanolic medium of these wines was in contact with the grapes’ skin and seeds was longer than the one corresponding to the wines produced under a temperature gradient. Such difference in liquid medium-contact time, together with their different liquid medium composition was expected to have an influence on the extraction process outcome.

### 3.1. Total Acidity and Ethanol Content Levels

Table 1 shows the average values of regular wine parameters. Two way ANOVA was applied to determine the differences resulting from the implementation of different maceration and fermentation temperatures. No significant interactions between these two variables were detected. It can be seen that the differences were only related to the total acidity levels of the wines obtained under different fermentation temperatures. On the other hand, the pre-fermentation maceration at 10 °C or 20 °C does not seem to affect the values of the main components (ethanol, pH, and total acidity), as no significant differences were found with respect to these parameters.

In terms of total acidity, the wines that had been fermented under a temperature gradient presented lower acidity levels than those fermented at a constant temperature (6.95 vs. 8.65 and 6.92 vs. 8.62) (Table 1). This is explained by the lower solubility of tartaric salts at low temperature. In the case of the fermentation under a temperature gradient, where the musts spent between 9 and 14 days at 10 °C a considerable precipitation of the tartaric salts would occur during that period at low temperature. This difference in total acidity did not affect the wines’ pH values. It has been described that the precipitation of tartaric salts does not produce any changes neither in the must pH level nor in that of the wines with pH values close to 3.6 [21].

The production of ethanol during the fermentation is related to the available biomass. The temperature during fermentation affects the level of biomass [18]. Therefore, the temperature during the fermentation conditions the production of ethanol. It has been reported that higher the temperature the lower the production of ethanol from the same initial level of sugars [22]. However, the lack of impact of the fermentation temperature on the final alcoholic strength has also been described. According to this, even low fermentation temperatures have no effect on the final alcohol content as long as the temperature is high enough to allow the development of the yeast. Thus, as can be seen in Table 1, the fermentation temperature did not have a clear influence on the final ethanol content, which means that the yeast was growing in the right conditions, even when the temperature was at the lowest level in the experiment. This poor impact on the final alcoholic strength has also been reported in the literature, according to which, even low fermentation temperatures have no effect on the final alcoholic strength, always provided that the temperature is high enough to support the viability of the yeasts [23].

Thus, with regard to their main components, the differences between the eight wines produced in this study were solely due to the specific conditions at the beginning of the alcoholic fermentation and the only effect that this variable had on the final wines was the already mentioned differences in total acidity.

### 3.2. Total Anthocyanins, Total Phenolics, and Total Tannins Contents

**Figure 2:**
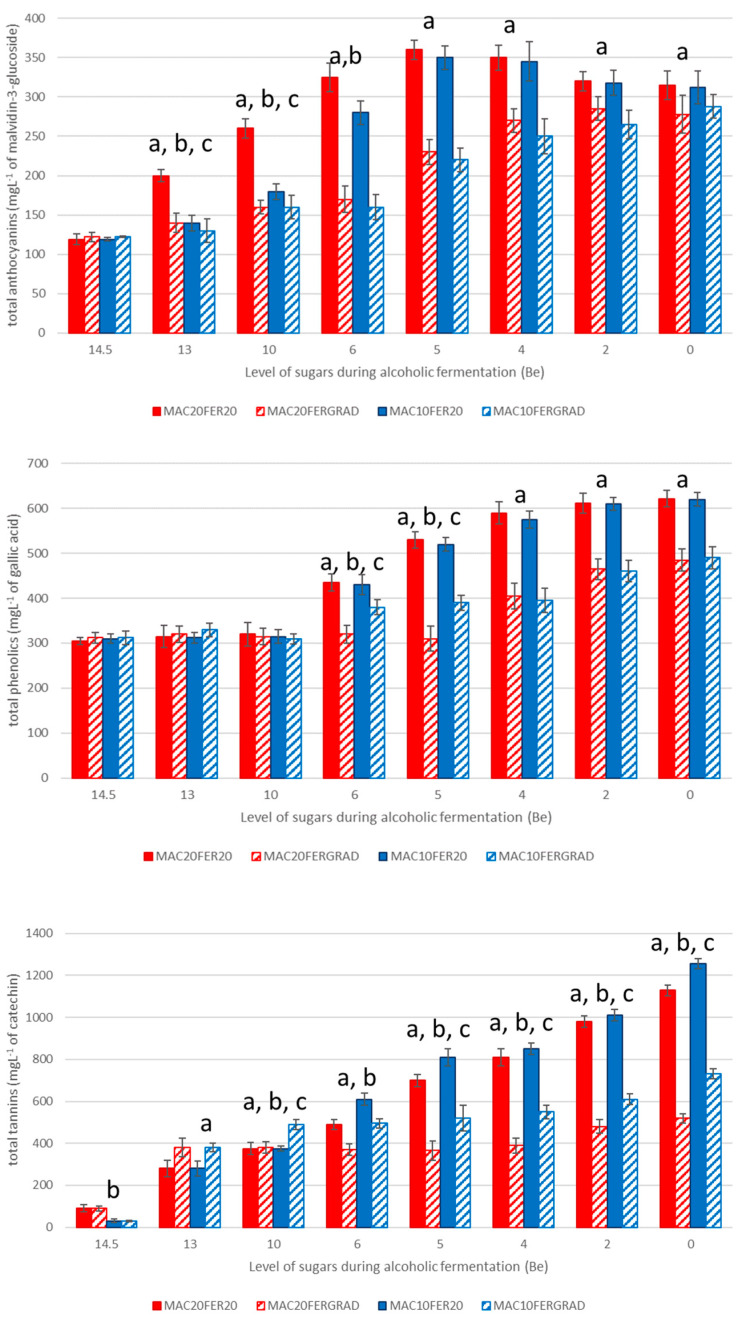
Evolution of the total content of anthocyanins (expressed as mg L^−1^ of malvidin-3-g Lucoside), phenolics (expressed as mg L^−1^ of gallic acid) and tannins (expressed as mg L^−1^ of catechin) over the fermentation processes under different conditions. a: significant differences due to changes in the fermentation temperature, b: significant differences due to changes in the maceration temperature, c: significant interactions between the maceration temperature and the fermentation temperature based on the ANOVA results (*p* < 0.05).

Figure 2 shows the evolution of total anthocyanins (expressed as mg L^−1^ of malvidin-3-g Lucoside), total phenolics (expressed as mg L^−1^ of gallic acid) and total tannins (expressed as mg L^−1^ of catechin) contents throughout the fermentation processes.

First of all, it should be noted that the final anthocyanins and polyphenols contents in the wines were very similar between those wines produced under the same fermentation temperature conditions, regardless of the temperature conditions during the pre-fermentative maceration (similar values of the striped and solid bars). These were unexpected values, particularly regarding anthocyanins, which are more soluble in must. In fact, it had been previously reported that pre-fermentative cold soaks produce higher levels of anthocyanins contents in must, particularly depending on the soaking temperature [24]. Therefore, larger contents should be expected in those wines produced through pre-fermentative maceration at 20 °C. In order to explain the results obtained in our study, it should be noted that during the pre-fermentative maceration the musts were not shaken at all and, therefore, it could be assumed that this lack of movement of the musts would result in a poorer extraction. It is worth mentioning that even the differences that had appeared during the first stages of the alcoholic fermentation (b in the bar graphics) had nearly disappeared at the end. It should also be pointed out that in our study, the free anthocyanins were the ones to be measured, and therefore the degradation and condensation of the tannins was also to be taken into account. In this regard, we should bear in mind that the highest levels of tannins were registered at the final stages of the alcoholic fermentation. We should also mention that it has been previously reported that the effects of pre-fermentative maceration on the resulting wine are conditioned by the specific grape variety [25].

In the case of the maceration, the development of the extraction process of the anthocyanins from the grapes into the musts did not show any variations regardless of the temperature. However, during the fermentation, the extraction of the anthocyanins proved to be dependent on the specific conditions [26]. Thus, when the musts were fermented at 20 °C (solid bars), a very rapid extraction rate was observed at the beginning of the alcoholic fermentation and later on, after the sugar level reached 5 °Be, it remained stable. A substantially slower extraction rate was registered at the first stages of the fermentation of the musts fermented under a temperature gradient (striped bars). This rapid extraction of anthocyanins at the early stages of the alcoholic fermentation had been previously reported, with fast extraction rates observed over the first 3–4 days [27]. In our experiment, when the fermentation temperature reached 20 °C, the content of anthocyanins increased gradually until very similar final levels were reached for all the wines, with 313 mg L^−1^ in the musts macerated at 20 °C and an average level of 309 mg L^−1^ malvidin-3-g Lucoside in the musts that started the maceration process at 10 °C. Therefore, the differences that had been registered at the beginning of the fermentation process were compensated by the faster extraction rate that was observed from the moment that the wines which had been fermented under a temperature gradient reached the maximum fermentation temperature.

Regarding the total phenolic content, obvious differences were registered while sugar levels were below 6 °Be. It should be noted that from then on, the fermentation temperature would gradually increase from 10 to 15 °C. No clear differences between stable-temperature and gradient-temperature wines were registered while in the lower temperature range, however, when the musts were fermented at constant higher temperatures (solid bars), they exhibited a visibly larger phenolic content. On the other hand, no differences were found at the end of the alcoholic fermentation that could be associated to pre-fermentative maceration conditions.

With respect to tannins content, it evolved differently from that of anthocyanins or total phenolics. Some marginal differences were found at the first stages of the alcoholic fermentation. The ANOVA results showed differences at the very first stages of the fermentation because of the previous differences during the pre-fermentative maceration conditions (Figure 2). Higher values were detected in the must that been maintained at 20 °C throughout the pre-fermentative maceration process (red bars). Once the fermentation started and sugar content reached 13 °Be, significant differences in tannins content started to appear. Thus, at higher sugar content levels, it was evident that temperature, either during the pre-fermentative maceration or during the fermentation, would condition the content levels of tannins in the must. Regarding the effect specifically attributable to the pre-fermentative maceration, it could be seen that the lower the temperature, the higher the content level of tannins in the must. No reason for this phenomenon was found to explain such greater tannins’ recoveries associated to lower temperatures during the pre-fermentative stage. Even more, when tannins contents in all the musts that had completed the pre-fermentative maceration process were low in all the cases. Therefore, other factors should be considered to enlighten these results. In this regard, a greater extraction of other compounds such as peptides or protein-related compounds during the pre-fermentative maceration could be suggested as a plausible explanation. Those extracted compounds would precipitate together with the tannins during the alcoholic fermentation, which would result in the subsequent lower tannins content in the final wine.

To sum up, the final values were 1256.7 ± 6.6 mg L^−1^ catechin in wines Ma10F20 versus 1129.3 ± 10.1 mg L^−1^ catechin in wines Ma20F20, and 732.2 ± 8.8 mg L^−1^ catechin in wines Ma10FGrad versus 519.5 ± 5.9 mg L^−1^ catechin in wines Ma20FGrad.

### 3.3. Red Color

Research studies in the literature support that pre-fermentative maceration could result in a wine with a deeper red color [28,29]. However, there are also published papers reporting limited effects of the pre-fermentative maceration on the final color of the wine [30]. In relation to this aspect, the evolution of a wine’s color during ageing and after the fermentation phase has been completed seems to be an interesting factor to be evaluated. It is well known that the evolution of wine color is related to its content in both anthocyanins and tannins.

**Figure 3:**
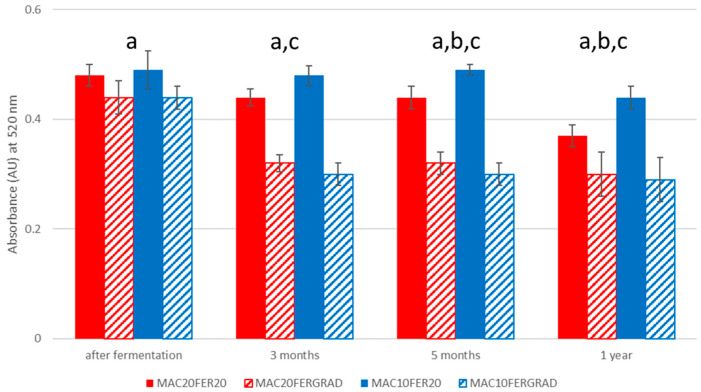
Evolution of the red color intensity of one-year-ageing wines produced through four different processes. a: significant differences due to differences in the fermentation temperature, b: significant differences due to differences in the maceration temperature, c: significant interaction between the maceration temperature and the fermentation temperature (*p* < 0.05).

During this study the typical evolution of wine color from a red to a reddish-brown hue due to oxidation could be observed. At the beginning of the ageing process, no differences because of the different pre-fermentative maceration conditions were observed, while the differences in the fermentation temperature resulted in evident differences between the four wines. Consequently and regardless of their pre-fermentative maceration temperature, the four wines exhibited color characteristics that would allow their classification based on their fermentation temperature (Figure 3). However, as time went by, the wines that had started their fermentation at 10 °C (Ma20FGrad and Ma10FGrad) suffered a more severe loss of color than those wines which had started their fermentation at 20 °C (Ma20F20 and Ma10F20) [31].

The role of the tannins in red wine with regard to its red color stability has already been described in the literature [32,33]. The results in Figure 3 demonstrate that the wines fermented at high temperature would show a higher color intensity than those produced under a temperature gradient. It seems clear that when the wines have been fermented under a temperature gradient they suffer a dramatic decrease in their color intensity after three months of ageing and from then on, their color remains stable. The loss in red color intensity was less dramatic, though steady, in those wines that had been produced under a constant fermentation temperature (solid bars). After five months of ageing, the effects of the pre-fermentative maceration appeared. The ANOVA results illustrate the effect of the interaction between the pre-fermentative and the fermentation conditions, as well as some significant differences attributable to just the pre-fermentative conditions. For example, the wines that had been produced at constant 20 °C over the pre-fermentative and the alcoholic fermentation presented lower absorbance values than those which had been macerated at constant 10 °C and then fermented at 20 °C (red solid bars vs. blue solid bars). It must also be noted that the wines subjected to pre-fermentative maceration at low temperature and fermentation at a constant temperature showed the highest red color intensity after ageing for one year. All of this seemed to indicate that the pre-fermentative maceration conditions may affect the stability of the red color intensity of the final wines. However, no color-loss effect associated to pre-fermentative maceration conditions could be detected during the ageing of those wines that had been fermented under a temperature gradient. Therefore, although the pre-fermentation conditions do not seem to have any influence on the extraction yields, they apparently facilitate the release of some compounds present in the grapes’ solid parts as long as the adequate fermentation conditions are applied.

### 3.4. Tasting Panel

**Figure 4:**
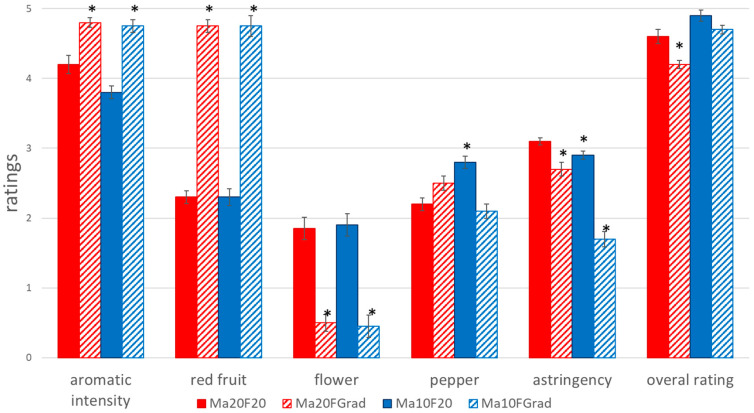
Assessment by the tasting panel. Bars accompanied by an asterisk (*) indicate a relevant sensory difference with respect to Ma20F20 wine (*p*-value < 0.05).

The descriptive analysis confirmed that the specific treatment of the musts and wines had noticeable effects on the aroma of the final product in terms of its fruity, pepper or floral characteristics as well as on its astringency. A particular wine sample that had been produced under high pre-fermentative maceration temperature and constant fermentation temperature, i.e., Ma20F20 was used as the reference for statistical comparison (Figure 4).

A more intense fruity aroma was associated to winemaking under fermentation temperature gradients (Ma20FGrad and Ma10FGrad), while Ma20F20 and Ma10F20 wines were perceived as having the lowest intensity in fruity aromas. These results are consistent with those from other research studies involving low-temperature fermentation processes that resulted in more aromatic wines [34,35]. On the other hand, Ma20F20 and Ma10F20 wines had a significantly higher “floral” aroma intensity than Ma20FGrad and Ma10FGrad wines. With regard to astringency, Ma20FGrad was granted by the judges significantly higher scores than those awarded to Ma10FGrad.

In terms of wine overall ratings, those that had undergone pre-fermentative maceration at 10 °C, and in particular Ma10F20 wines were awarded the best tasting scores, even if no significant differences were registered with respect to those assigned to Ma20F20 wines.

## 4. Conclusions

Temperature conditions during the pre-fermentative maceration of red grape musts seems to have hardly any effect on the final wines. On the other hand, temperature levels during the alcoholic fermentation process have a noticeable influence on the tannins content of the final product. Thus, the implementation of a temperature gradient during the alcoholic fermentation results in wines with a lower tannin content. Furthermore, when wines are produced under a temperature gradient their red color seems to be less stable. In fact, it has been proven that after 1 year in the bottle, the average loss of red color intensity can reach up to 28% in those wines that have been fermented under a temperature gradient, compared to the 13% fall in color intensity that characterizes wines fermented at a constant temperature. On the other hand, together with a lighter color, wines subjected to a temperature gradient during their fermentation, exhibit a more intense fruity aroma.

In our experiment, given that all the wines started their alcoholic fermentation with similar red color intensity levels, their final color intensity could solely be attributed to the amount of tannins that were extracted over the alcoholic fermentation phase.

Further studies on the effect of other temperature gradients (for example, higher temperatures at the final stages) that may render similar results with regards to aroma properties while maintaining higher tannins content levels might be advisable.

## Figures and Tables

**Table 1:** Average acidity, pH level and ethanol content of the wines produced under different winemaking conditions.

	Total Acidity (g L^−1^ of Tartaric Acid) ^a^	pH	Ethanol (%)
**Ma20F20**	8.62 ± 0.09	3.60 ± 0.10	14.54 ± 0.18
**Ma20FGrad**	6.92 ± 0.06	3.63 ± 0.06	14.41 ± 0.19
**Ma10F20**	8.65 ± 0.07	3.58 ± 0.12	14.61 ± 0.17
**Ma10FGrad**	6.95 ± 0.05	3.69 ± 0.06	14.69 ± 0.19

^a^: significant differences due to the changes in fermentation temperature (*p* < 0.05).

## Data Availability

Data available on request.
